# Surgical management of small bowel metastasis from esophageal squamous cell carcinoma: a case report and literature review

**DOI:** 10.3389/fsurg.2026.1802310

**Published:** 2026-09-07

**Authors:** Qiujin Huang, Xiang Huifei, Liu Qiaoyun

**Affiliations:** Department of Gastrointestinal Surgery, General Hospital of Hunan University of Medicine, Huaihua, China

**Keywords:** case report, esophageal squamous cell carcinoma, intestinal obstruction, lymphatic dissemination, molecular biomarkers, palliative surgery, small bowel metastasis

## Abstract

**Background:**

Small bowel metastasis from esophageal squamous cell carcinoma (ESCC) is an exceedingly rare event, typically diagnosed only during emergency surgery for obstruction or perforation. The underlying metastatic mechanisms and optimal management strategies remain poorly defined.

**Case presentation:**

A 54-year-old woman with stage IVB ESCC (cT3N3M1; left supraclavicular lymph node metastasis) developed recurrent complete intestinal obstruction while receiving systemic chemo-immunotherapy and local radiotherapy. Emergency laparotomy revealed multiple serosal small bowel metastases causing segmental stenosis. Approximately 20 cm of jejunum was resected with primary side-to-side anastomosis, and a decompressive tube jejunostomy was placed. The postoperative course was uneventful, and the obstruction resolved. However, due to a rapid decline in performance status, no further anticancer therapy could be administered, and the patient died of cachexia and electrolyte imbalance two months after surgery.

**Conclusions:**

In patients with known esophageal cancer, the development of “atypical” small bowel obstruction should raise immediate suspicion for peritoneal metastasis, even when conventional imaging fails to demonstrate discrete masses. Prompt surgical exploration can achieve both definitive diagnosis and effective palliation. This case also highlights the probable lymphatic route of metastatic spread and underscores the urgent need to integrate molecular biomarkers into surveillance strategies for rare but lethal metastatic patterns.

## Introduction

Esophageal squamous cell carcinoma (ESCC) remains a major global health burden, particularly in Asia. In 2022, over 511 000 new cases and 445 000 deaths were reported worldwide, with China accounting for a disproportionately high share of the incidence (1). The dominant metastatic routes are lymphatic dissemination and, to a lesser degree, hematogenous spread to the liver, lungs, and bones. Metastasis to the small bowel is exceedingly rare: fewer than 100 surgically confirmed cases have been documented since 1985 (2–4), and the vast majority of small bowel metastases are adenocarcinomas of colorectal or gastric origin; squamous cell carcinomas account for less than 1% of all small bowel metastases (5).

We report a case of ESCC that metastasized to the small bowel during systemic therapy, causing recurrent complete intestinal obstruction. By presenting the clinical decision-making process, intraoperative findings, and outcome, and by critically discussing potential metastatic pathways, we aim to raise awareness of this rare entity and provide a reference for multidisciplinary management. We also discuss the limitations of current diagnostic modalities and emphasize the future role of molecular profiling in predicting uncommon metastases.

## Case presentation

**Figure 1:**
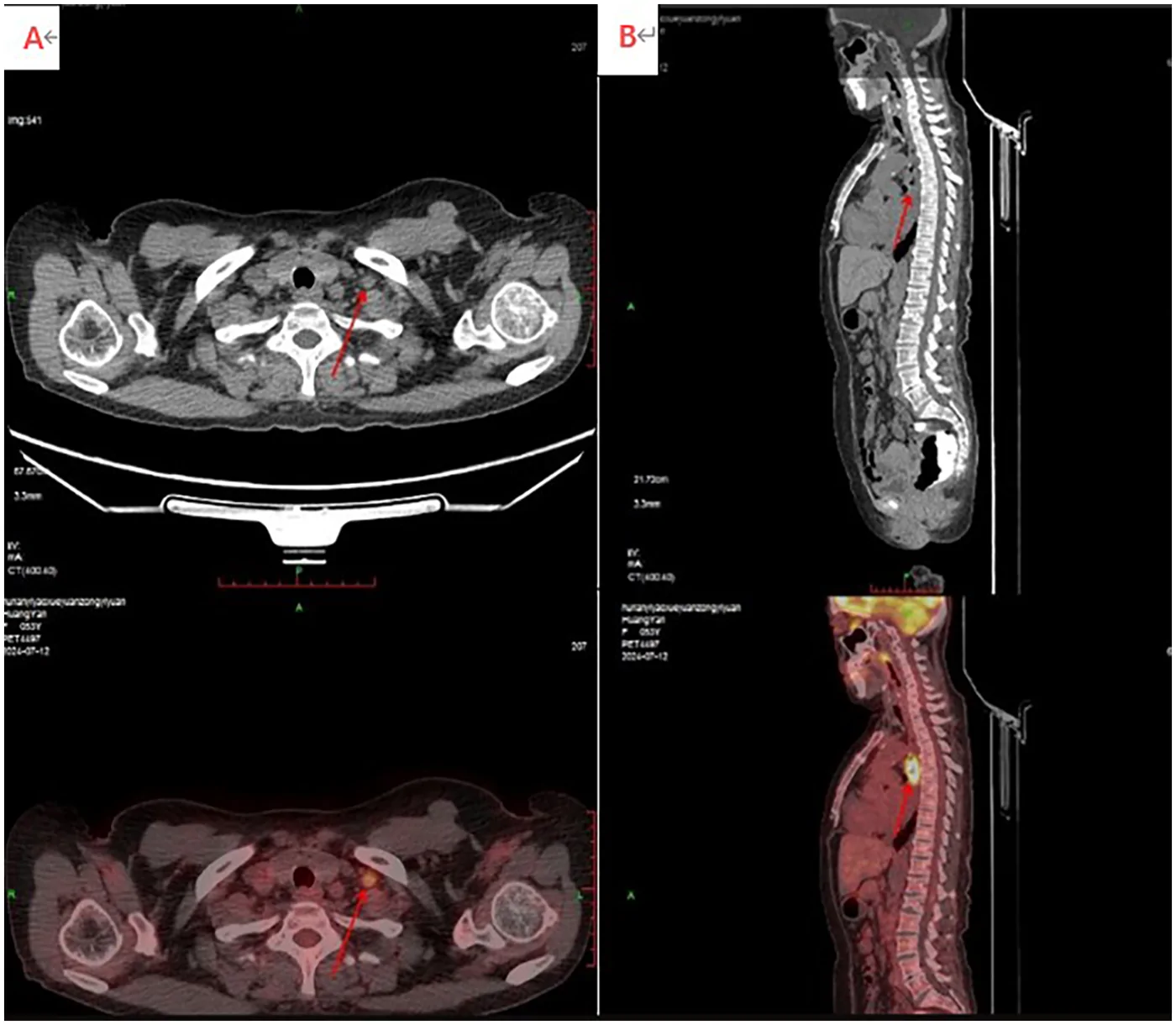
Histopathology of the primary esophageal tumor. **(A)** Low-power view (  ×  2, H&E) showing nests of squamous cell carcinoma. **(B)** High-power view (  ×  20, H&E) demonstrating keratin pearls and marked cellular atypia.

**Figure 2:**
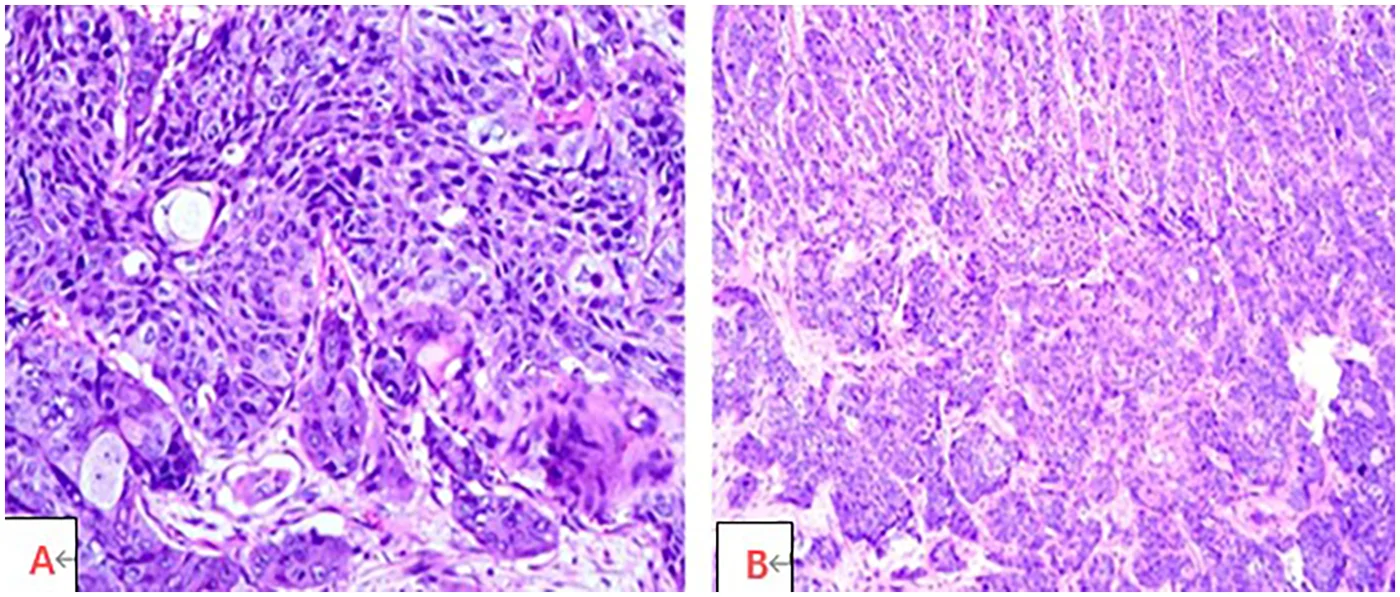
^1^⁸F-FDG PET-CT images. **(A)** Hypermetabolic focus in the left supraclavicular lymph node (arrow). **(B)** Hypermetabolic focus in the upper thoracic esophagus (arrow).

**Figure 3:**
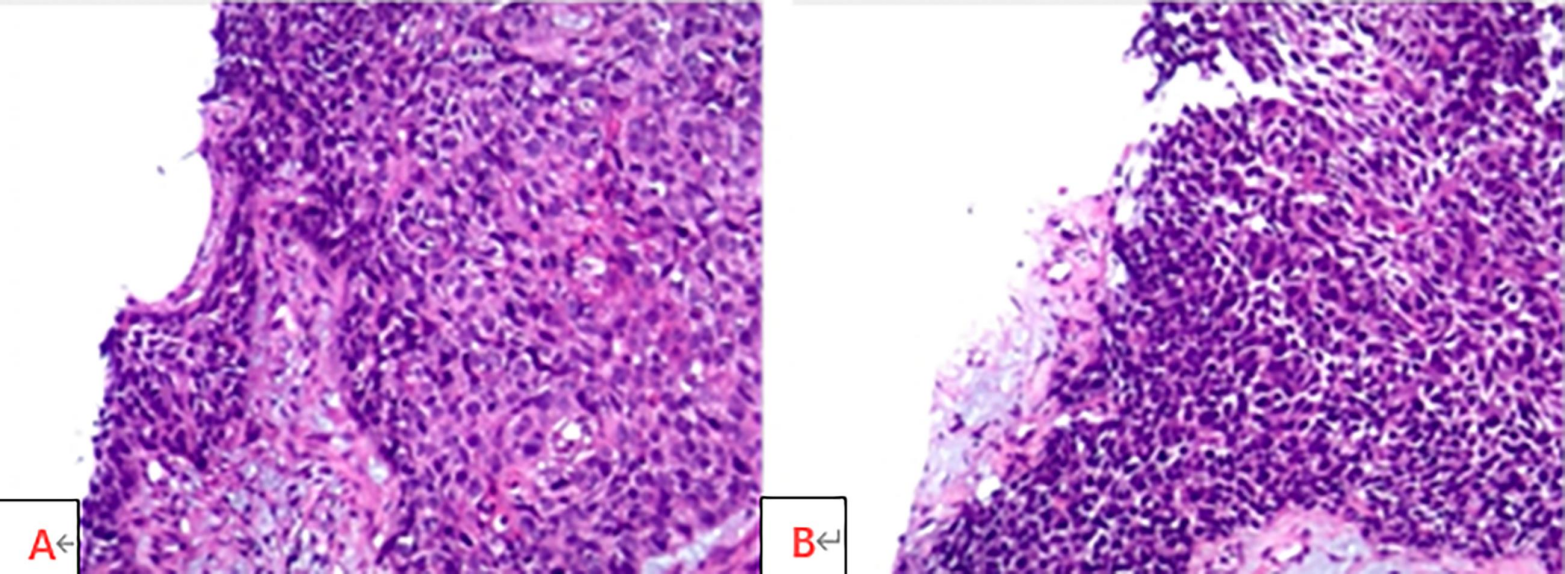
Needle biopsy of the left supraclavicular lymph node. **(A)** Low-power view (  ×  2, H&E) showing metastatic squamous cell carcinoma within the lymph node. **(B)** High-power view (  ×  20, H&E) demonstrating infiltrating tumor cells.

A 54-year-old woman presented in July 2024 with a four-month history of progressive dysphagia, a choking sensation, and two days of hematemesis. She had no family history of malignancy and was a lifelong non-smoker. Physical examination revealed a firm, fixed left supraclavicular lymph node (approximately 1.5 cm × 1.0 cm); the abdomen was flat, soft, and non-tender, with normal bowel sounds. Esophagoscopy disclosed an irregular stenotic mass located 21–26 cm from the incisors; biopsy confirmed moderately differentiated squamous cell carcinoma (Figure 1). Integrated ^1^⁸F-FDG PET-CT demonstrated hypermetabolic activity in both the primary tumor and the left supraclavicular lymph node (Figure 2), and needle biopsy of the node confirmed metastatic squamous cell carcinoma (Figure 3). The clinical stage was cT3N3M1, stage IVB, according to the 8th edition of the AJCC staging system.

Because the patient was not a candidate for curative resection, PD-L1 expression was assessed according to contemporary CSCO guidelines, yielding a combined positive score (CPS) ≥ 10. From July to September 2024, she received three cycles of tislelizumab combined with nab-paclitaxel and cisplatin, with transient improvement in dysphagia. However, in October 2024, complete esophageal obstruction recurred. To ensure nutritional access, ultrasound-guided percutaneous gastrostomy with a jejunal extension tube was successfully placed, and enteral nutrition was well tolerated. Given that the disease was oligometastatic (only the left supraclavicular node), high-dose palliative radiotherapy to the primary tumor was initiated on 25 October 2024 to achieve local control and symptom relief.

**Figure 4:**
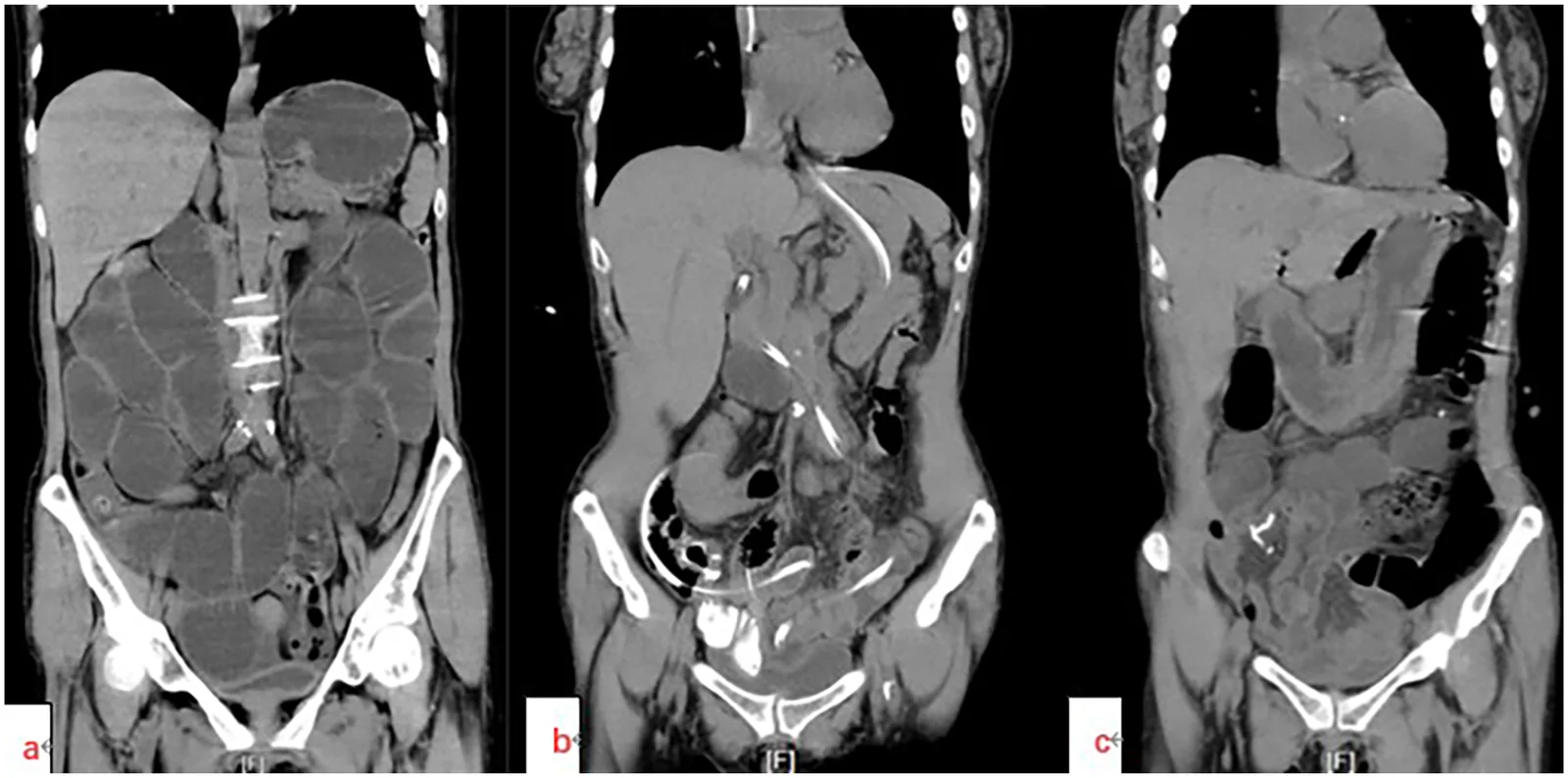
Contrast-enhanced abdominal CT scans. **(a)** At the first episode of obstruction (November 2024): dilated small bowel loops with a transition point. **(b)** Obstruction recurrence after tube removal (December 2024): persistent small bowel obstruction. **(c)** Resolution of obstruction after surgical resection (December 2024).

Near the end of the radiotherapy course, on the night of 4 November 2024, the patient suddenly developed severe periumbilical cramping pain, abdominal distension, and absolute constipation. Physical examination revealed abdominal distension without visible peristalsis, mild periumbilical tenderness without rebound, and hyperactive, high-pitched bowel sounds. Emergency contrast-enhanced computed tomography (CT) of the abdomen demonstrated small bowel obstruction with a transition point in the mid-abdomen; no discrete mass was identified (Figure 4a). Given the patient's history of appendectomy, adhesive obstruction was initially suspected. A long nasointestinal tube was inserted, which drained a large volume of intestinal content and led to dramatic symptomatic relief, with prompt return of flatus and stool passage. The tube was removed on 1 December 2024.

Three days later, however, the symptoms of complete obstruction recurred, and repeat CT again showed small bowel obstruction at the same location (Figure 4b). Conservative measures failed to resolve the obstruction. After multidisciplinary discussion, it was concluded that the rapid recurrence was inconsistent with simple adhesive obstruction, and occult peritoneal metastasis could not be excluded. Although imaging did not reveal a discrete mass, the complete obstruction refractory to conservative treatment constituted an indication for surgical exploration.

**Figure 5:**
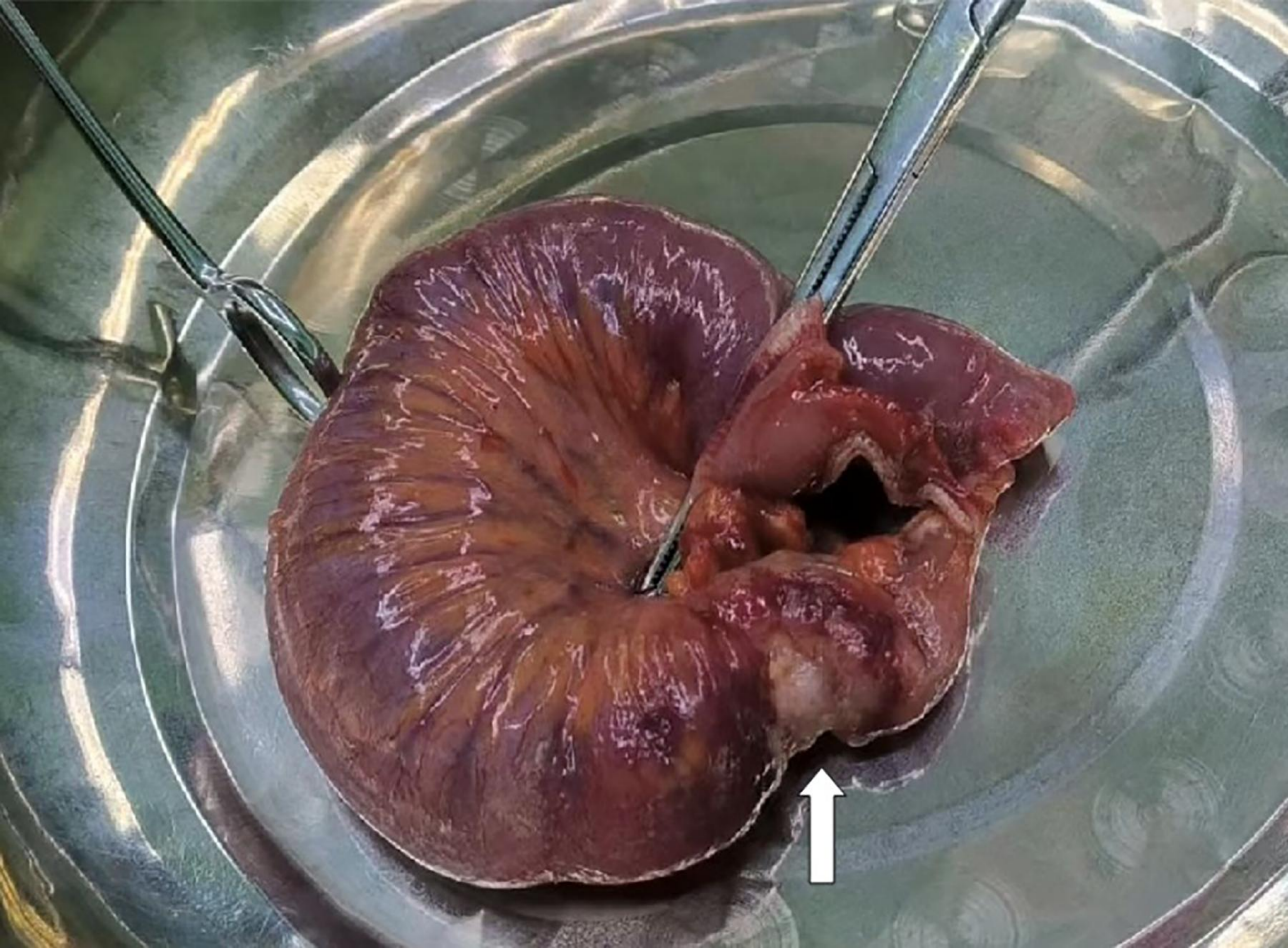
Resected small bowel specimen. The white arrow indicates the main stenotic lesion located approximately 60 cm from the ileocecal valve.

**Figure 6:**
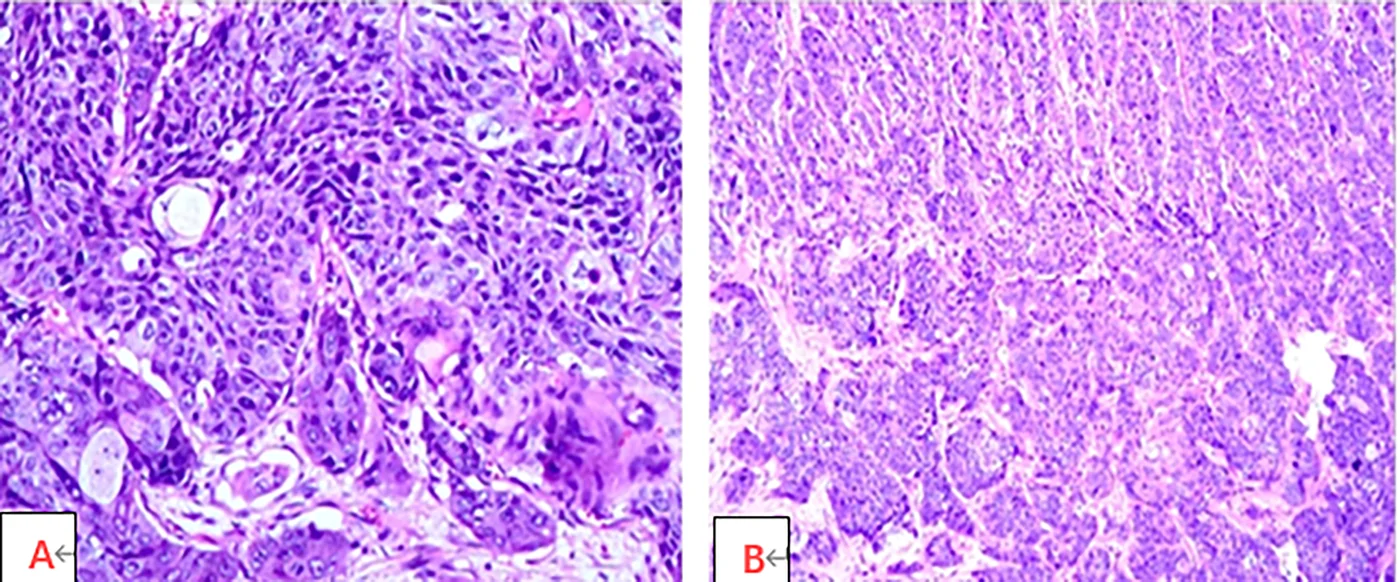
Histopathology of the small bowel metastasis. **(A)** Low-power view (  ×  2, H&E) showing full-thickness infiltration by tumor. **(B)** High-power view (  ×  20, H&E) showing squamous differentiation with keratin pearl formation.

On 21 December 2024, an emergency exploratory laparotomy was performed. A small amount of clear ascites was present, and no adhesive bands were identified. Systematic inspection of the entire small bowel revealed five gray-white, hard subserosal nodules. A protruding mass (2.5 cm × 2 cm × 1 cm) located approximately 60 cm from the ileocecal valve caused segmental luminal stenosis, and a second stenotic ring was found 13 cm distal to this lesion (Figure 5). The lesions were interpreted as multiple small bowel metastases, with the more proximal mass being the direct mechanical cause of the obstruction. A segmental resection of approximately 20 cm encompassing both main lesions was performed, followed by a side-to-side small bowel anastomosis. Because multiple macroscopic metastatic nodules remained unresected within the peritoneal cavity, a Witzel-type tube jejunostomy was fashioned proximal to the anastomosis to secure postoperative enteral nutrition. Histopathological examination of the resected specimen confirmed metastatic squamous cell carcinoma with full-thickness invasion into the subserosa; morphological and immunohistochemical features (CK5/6+, p40+) were identical to those of the primary esophageal tumor (Figure 6).

**Figure 7:**
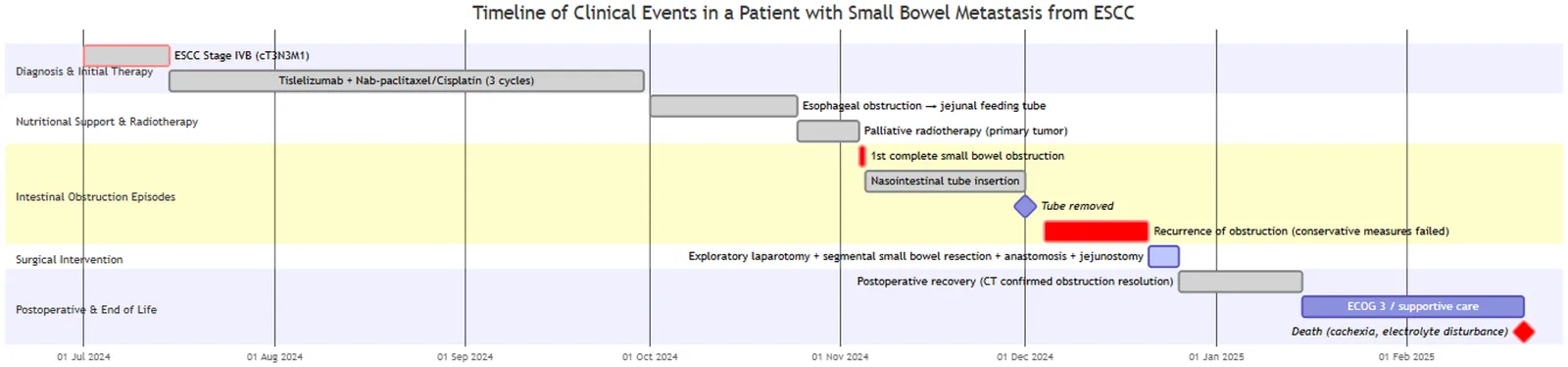
Graphical timeline of the clinical course. The timeline illustrates the sequence of major events from initial diagnosis in July 2024 to death in February 2025, including oncologic therapies, episodes of obstruction, tube insertion, surgery, and outcome. Overall survival was approximately 7 months.

The postoperative course was uneventful, with no anastomotic leak, wound infection, or other complications. A CT scan on postoperative day 5 confirmed complete resolution of the obstruction (Figure 4c), and enteral feeding was successfully established via the jejunostomy tube. Nevertheless, the patient's performance status declined to ECOG 3, precluding further anticancer therapy. She succumbed to cachexia and refractory electrolyte disturbances caused by progressive tumor dissemination in February 2025, two months after surgery.

The patient and her family had been fully informed about the limited curative potential and operative risks prior to surgery. They agreed to the procedure with the understanding that the primary aims were to obtain a definitive diagnosis and to palliate the obstruction. Written informed consent for publication of this case was obtained from the patient's next of kin following her death Table 1.

**Table 1:** Timeline of major clinical events. A graphical summary is provided in Figure 7.

Date	Key event	Remarks
Jul 2024	Dysphagia, hematemesis; ESCC diagnosis; cT3N3M1 stage IVB	Left supraclavicular node metastasis confirmed
Jul–Sep 2024	Three cycles of tislelizumab + nab-paclitaxel/cisplatin	CPS ≥ 10
Early Oct 2024	Severe esophageal obstruction; jejunal feeding tube placed	Enteral nutrition initiated
25 Oct 2024	High-dose palliative radiotherapy started	Aimed at oligometastatic local control
4 Nov 2024	First episode of complete small bowel obstruction	CT: mid-abdominal obstruction
5 Nov 2024	Long nasointestinal tube inserted; obstruction resolved	Conservative management effective
1 Dec 2024	Tube removed	–
4 Dec 2024	Recurrence of complete obstruction; conservative measures failed	CT unchanged
21 Dec 2024	Emergency laparotomy: multiple small bowel metastases; segmental resection + anastomosis + jejunostomy	Histology: metastatic SCC
26 Dec 2024	Postoperative CT: obstruction resolved	–
Jan–Feb 2025	ECOG 3; supportive care only	–
Feb 2025	Death from cachexia and electrolyte disturbances	Overall survival ∼7 months

## Discussion

Small bowel metastasis from ESCC is an uncommon but clinically significant event, typically presenting with obstruction, perforation, or hemorrhage (3, 4, 6). In this patient, the obstructive metastasis became clinically apparent while she was receiving systemic chemo-immunotherapy and local radiotherapy. This temporal relationship suggests that micro-metastatic deposits may already have been present at initial diagnosis, and that treatment-induced changes in the tumor microenvironment—such as mucosal injury exposing the basement membrane—could have facilitated the adhesion and proliferation of circulating squamous carcinoma cells (7, 8). Furthermore, the immunosuppressive effects of chemotherapy may have contributed to the escape of dormant tumor cells from immune surveillance, allowing rapid progression within the small bowel serosa.

Anatomically, the hematogenous route to the small bowel requires tumor cells to enter the portal venous system, which is physiologically separate from the systemic venous return in the absence of liver metastases. A more plausible pathway for squamous cell carcinoma is lymphatic dissemination. In **lower esophageal cancers**, the pattern of lymph node spread frequently involves the left gastric artery nodes, which can serve as a conduit to the mesenteric lymphatic network and ultimately reach the ileocecal region (9, 10). However, our patient's primary tumor was located in the **upper thoracic esophagus** (21–26 cm from the incisors), an area that primarily drains to cervical and upper mediastinal nodes—as evidenced by the prominent left supraclavicular metastasis at presentation. In **upper esophageal cancers**, the thoracic duct provides direct drainage into the left subclavian vein, which can seed tumor cells into the systemic circulation and thus bypass the portal system. Additionally, extensive retrograde lymphatic flow can occur when proximal drainage is impeded, potentially disseminating tumor cells to the peritoneal cavity and small bowel serosa via transdiaphragmatic lymphatic channels. Therefore, the presence of a left supraclavicular node metastasis at diagnosis likely indicated early breach of the central lymphatic barrier, which subsequently led to widespread peritoneal and small bowel involvement. This case illustrates that **upper esophageal cancers can metastasize to the small bowel via a combination of systemic lymphatic dissemination and possibly direct peritoneal seeding, rather than through the left gastric artery route characteristic of distal esophageal tumors**. This distinction has not been adequately emphasized in previous reports and addresses an important gap in understanding metastatic patterns for mid/upper esophageal SCC.

An important clinical lesson highlighted by this case is the limitation of conventional imaging. Even with contrast-enhanced CT, the small bowel metastases appeared only as non-specific obstruction without identifiable mass lesions. A high index of suspicion is therefore required when a patient with known advanced esophageal malignancy presents with “atypical” intestinal obstruction—particularly when the obstruction recurs rapidly after initial conservative management. In such scenarios, diagnostic laparoscopy or exploratory laparotomy should not be delayed, as surgical exploration can simultaneously establish a tissue diagnosis and provide effective palliation. Recent advances in whole-body diffusion-weighted magnetic resonance imaging (WB-DWI/MRI) and ^1^⁸F-FDG PET-CT with oral contrast may improve the detection of small peritoneal implants, but these modalities were not utilized in this case due to clinical urgency.

This study has several notable limitations. First, as a single retrospective case report, the findings cannot be generalized. Second, because the patient's condition deteriorated rapidly, we were unable to perform comparative molecular profiling of the primary and metastatic lesions; testing for clinically relevant biomarkers such as EGFR amplification, FGFR pathway activation, PD-L1 expression, and tumor mutational burden (TMB) could have provided mechanistic insights into the metastatic process and potentially identified therapeutic targets. Third, quality-of-life assessments and patient-reported outcomes were not systematically collected, a gap that should be addressed in future prospective studies.

Looking forward, the integration of liquid biopsies and multi-omics analyses into routine surveillance for high-risk ESCC patients holds great promise. Emerging evidence suggests that circulating tumor cells (CTCs), circulating tumor DNA (ctDNA), and exosomal microRNAs can predict metastatic potential before radiological evidence appears (11). Specific molecular alterations, such as TP53 mutation, PIK3CA amplification, and chromosomal instability, are increasingly recognized as drivers of peritoneal metastasis in gastrointestinal cancers. Moreover, the tumor microenvironment—including cancer-associated fibroblasts (CAFs) and the extracellular matrix composition of the primary tumor—may influence the tropism of metastatic cells for the small bowel (12). Future prospective studies should systematically collect fresh biopsy samples from both primary and metastatic sites to perform whole-exome sequencing, transcriptomic analysis, and immune profiling, with the goal of constructing molecular prediction models for rare metastatic patterns.

## Conclusion

This case demonstrates that ESCC can metastasize to the small bowel and cause life-threatening complete intestinal obstruction, even when radiological imaging fails to reveal discrete lesions. When conservative treatment fails, prompt surgical exploration and palliative segmental resection should be considered to relieve symptoms and restore enteral nutrition. The most plausible mechanism in upper esophageal cancer appears to involve systemic lymphatic dissemination through the thoracic duct and possibly retrograde peritoneal lymphatic flow, rather than the left gastric artery route typical of distal tumors. Beyond surgical management, the present case underscores the urgent need for integrating molecular biomarkers—such as circulating tumor DNA assays, next-generation sequencing of metastatic tissue, and immune profiling—into future surveillance programs. Such precision oncology approaches may one day enable the early prediction and interception of rare but universally fatal metastatic patterns like small bowel involvement, ultimately improving outcomes for patients with advanced esophageal cancer.

## Data Availability

The raw data supporting the conclusions of this article will be made available by the authors, without undue reservation.
